# Supplementary material for: Cellulosic Ethanol Production Using Waste Wheat Stillage after Microwave-Assisted Hydrotropic Pretreatment
Source: Molecules. 2022 Sep 18;27(18):6097. doi: 10.3390/molecules27186097 (PMC9506164; doi:10.3390/molecules27186097)
Supplement: Supplementary file 1 [file molecules-27-06097-s001.zip › molecules-1872902-supplementary.pdf]

Supplementary Material

Table S1. Statistical analysis of variance (ANOVA).

Source	DF	Adj SS	Adj MS	F-value	P-Value
Model	26	4929.0	189.6	31.31	<0.001*
Enzyme	2	3205.5	1602.8	264.69	<0.001*
Biomass	2	195.7	97.9	16.16	<0.001*
Time	2	1398.1	699.0	115.44	<0.001*
Enzyme*Biomass	4	35.1	8.8	1.45	0.231
Enzyme*Time	4	73.1	18.3	3.02	0.026*
Biomass*Time	4	13.3	3.3	0.55	0.702
Enzyme*Biomass*Time	8	8.3	1.0	0.17	0.994
Error	54	327.0	6.1		

R-sq = 0.9378.

DF-Degree of freedom; Adj SS-Adjusted sum of squares; Adj MS-Adjusted mean squares; P-Value-Probability; F-Value-Test statistics; S-significant * $p < 0.05$.
